# Reduced response to SARS-CoV-2 vaccination is associated with impaired immunoglobulin class switch recombination in SLE patients

**DOI:** 10.1093/cei/uxae119

**Published:** 2024-12-10

**Authors:** Guillem Montamat, Claire E Meehan, Hannah F Bradford, Reşit Yıldırım, Francisca Guimarães, Marina Johnson, David Goldblatt, David A Isenberg, Claudia Mauri

**Affiliations:** Division of Infection and Immunity and Institute of Immunity and Transplantation, Royal Free Hospital, University College London, London, UK; Division of Infection and Immunity and Institute of Immunity and Transplantation, Royal Free Hospital, University College London, London, UK; Division of Infection and Immunity and Institute of Immunity and Transplantation, Royal Free Hospital, University College London, London, UK; Centre for Rheumatology, Division of Medicine, University College London Hospital, London, UK; Centre for Rheumatology, Division of Medicine, University College London Hospital, London, UK; Great Ormond Street Institute of Child Health Biomedical Research Centre, University College London, London, UK; Great Ormond Street Institute of Child Health Biomedical Research Centre, University College London, London, UK; Centre for Rheumatology, Division of Medicine, University College London Hospital, London, UK; Division of Infection and Immunity and Institute of Immunity and Transplantation, Royal Free Hospital, University College London, London, UK

**Keywords:** systemic lupus erythematosus, vaccine, vaccination

## Abstract

**Introduction:**

Systemic lupus erythematosus (SLE) patients exhibit B-cell abnormalities. Although there are concerns about reduced antibody responses to SARS-CoV-2 vaccines, detailed data on B-cell-specific responses in SLE remain scarce. Understanding the responsiveness to novel vaccine antigens, and boosters number, is important to avoid unnecessarily prolonged isolation of immunocompromised individuals. We assessed humoral and antigen-specific B-cell subset responses, including changes in isotype switching, prior to and after several doses of SARS-CoV-2 vaccines.

**Methods:**

Blood samples were obtained prior to and after SARS-CoV-2 vaccination from cross-sectional and longitudinal cohorts of previously uninfected patients with SLE (*n* = 93). Healthy participants receiving SARS-CoV-2 vaccines were recruited as controls (*n* = 135). We measured serum antibody titres, their neutralizing capacity, and vaccine-specific memory B-cell subsets.

**Results:**

Impaired IgG, IgA, and neutralizing responses against the original and various SARS-CoV-2 variants were observed following two doses of vaccine in SLE patients. Follow-up booster doses increased humoral responses compared to baseline, but they remained lower, with poorer neutralisation capacity against most strains, compared to healthy individuals after three or more doses. Analysis of memory B-cell subsets in SLE patients revealed an increase of SARS-CoV-2-specific isotype unswitched IgM^+^ over SARS-CoV-2-specific isotype switched IgG^+^/IgA^+^ memory B-cells compared to healthy individuals. Culturing healthy naive B-cells with high levels of IFNα, a hallmark of SLE pathogenesis, prevented B-cells from switching to IgG under IgG-polarizing conditions.

**Conclusion:**

SLE patients’ protection against SARS-CoV-2 is overall impaired compared to healthy individuals and is associated with a class switch defect possibly due to chronic exposure of B-cells to IFNα.

## Teaser

SLE patients exhibit a lower antibody response to SARS-CoV-2 vaccination compared to healthy controls. SLE patients show reduced antibody class-switch recombination after SARS-CoV-2 vaccination compared to healthy controls. High concentrations of IFN-alpha impair class-switch recombination to IgG.

## Graphical Abstract

**Graphical Abstract:**
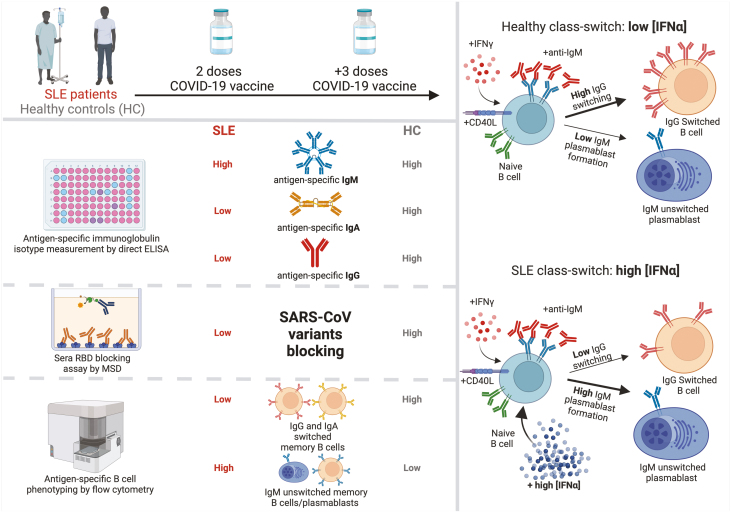

## Introduction

Systemic lupus erythematosus (SLE) is a chronic and clinically heterogeneous autoimmune disease, characterized by the presence of pathogenic autoantibodies. SLE patients present with profound B-cell abnormalities [1], including expansions of pathogenic CD27^−^IgD^−^ double-negative (DN) B cells, autoantibody-producing plasmablasts, and by reduced frequencies of anti-inflammatory regulatory B cells (Bregs) [2]. B-cell dysregulation is often associated to more severe disease and to high autoantibody titres.

During infection, early extrafollicular B-cell reactions, resulting in IgM production [3], provide initial protection against pathogens, followed by the generation of long-lasting B-cell immunity occurring through follicular activation including the germinal centre (GC) reaction [4]. GC formation and the consequent development of somatically hypermutated memory B cells and plasma cells are pivotal in driving efficacious protective immunity not only following infection but also following vaccination. Follicular responses facilitate class switch recombination (CSR), leading to the production of more functional IgG and IgA antibodies [5]. It has been previously reported that SARS-CoV-2 mRNA vaccination induces a persistent GC response that can last up to 6 months in the general population [6].

Comprehensive characterization of the antibody-mediated immune reaction in SLE patients, including both antibody quality and detailed B-cell response, remains largely unexplored [7–12]. To bridge this gap in knowledge, we compared the efficacy of SARS-CoV-2 vaccination in a cohort of SLE patients and healthy individuals by measuring serological and vaccine-specific B-cell responses.

In agreement with previous findings [7], we show a reduced response to SARS-CoV-2 vaccination following the initial two doses. Compared to healthy controls, SLE patients exhibit lower receptor-binding domain (RBD)- or spike protein subunit 1 (S1)-specific IgG titres with weaker neutralizing capacity. We reiterate the importance of booster vaccinations for immunocompromised individuals in building sufficient functional antibody responses. However, we report that SLE patients present a CSR defect in vaccine-specific B cells compared to healthy individuals as shown by an accumulation of vaccine-specific IgM^+^ unswitched memory B cells. We recapitulate the CSR defect by culturing healthy B cells with high levels of IFNα, a major contributor to B-cell abnormalities in SLE, under IgG isotype switching polarizing conditions. Taken together our results show that IFNα-induced defective CSR could underpin to sub-optimal immune response to vaccination in SLE patients.

## Methods and materials

### Study population

Peripheral blood samples for PBMC and serum isolation were collected from healthy donors and from SLE patients attending the University College London Hospital (UCLH) rheumatology outpatients’ clinic. Ethical approval was obtained from the UCLH Health Service Trust ethics committee, under REC reference no. 14/SC/1200. Healthy controls (*n* = 135) and patients (*n* = 93) were recruited following informed consent. Sample storage complied with the requirements of the Data Protection Act 1998. Demographics, clinical features, routine laboratory testing, and therapeutic regimen were collected retrospectively from electronic medical files. Dates of vaccination and history of SARS-CoV-2 infection were also recorded for healthy controls and SLE patients. Detailed exclusion criteria are described in Online Supplementary Material.

### Quantification of RBD-specific, S1-specific, and total IgG, IgA, and IgM titres

RBD- and S1-specific direct ELISA protocols were carried out as previously published [13] with modifications. Similarly, RBD-omicron-specific IgG was an adaption of this protocol using an anti-omicron IgG monoclonal antibody. RBD-omicron-specific IgA was determined using a commercial kit (RAS-T099, AcroBiosystems). To measure the total isotype (IgG, IgA, and IgM) immunoglobulin concentration in serum samples, a sandwich ELISA (88-50550-22 for IgG, 88-50600-88 for IgA, 88-50620-88 for IgM, Invitrogen) was performed according to the manufacturer’s instructions. Detailed protocols are described in the Supplementary Methods.

### Blocking assay

A V-PLEX SARS-CoV-2 panel 30 (ACE2) kit (K15635U, Meso Scale Diagnostics) was used according to the manufacturer’s instructions to measure the capacity of serum samples from SLE patients and healthy controls to block the binding of variants of SARS-CoV-2 to the ACE2 receptor. A dilution of 1:50 was used for all serum samples. Individuals with blocking capacity >50% for any tested SARS-CoV-2 strain were considered as neutralizers.

### *Ex vivo* B-cell phenotyping

SARS-CoV-2 antigen-specific B-cells were identified using labelled RBD (793604, Biolegend) and S1 (792906, Biolegend) proteins with the addition of antibodies against phenotypic markers and BCR isotype-specific antibodies to measure class-switch status. Full antibody panels and protocols used for *ex vivo* B-cell phenotyping are described in Supplementary Methods. Stained samples were acquired using a Cytek™ Aurora spectral cytometer, using SpectroFlo (v3.0.3) with automated unmixing. Data were analysed using FlowJo (TreeStar, v.10.8.1).

### IFNα CSR *in vitro* culture

Naive B cells were sorted from previously frozen PBMCs of healthy controls, using a BD FACSAria™ Fusion and BD FACSDiva (v9.4). Full details of cell sorting protocols are described in Supplementary Methods. Sorted naive B cells from healthy controls were cultured *in vitro* under IgG-polarizing class-switching conditions, consisting of complete RPMI (10% heat-inactivated foetal calf serum, 1% penicillin/streptomycin), 1 μg/ml anti-IgM (309-006-043, Jackson ImmunoResearch), 1 μg/ml CD40L (ALX-522-110-C010, Enzo Life Sciences), and 50 ng/ml recombinant human IFN*ˠ* (285-IF-100/CF, Bio-Techne), in a round-bottom 96-well plate at 37 ^o^C and 5% CO_2_ for 6 days. In addition, IFNα (11200-1, pbl assay science) was added at the indicated concentrations. IgG polarising class-switch medium without or with the indicated concentration of IFNα was refreshed on Day 3 by gently centrifuging the cells (300*g* for 10 minutes) and adding fresh media including the above-mentioned factors. Staining protocols and antibody panels used are described in Supplementary Methods. Stained samples were acquired using a Cytek™ Aurora spectral cytometer, using SpectroFlo (v3.0.3) with automated unmixing. Data were analysed using FlowJo (Treestar, v10.8.1).

### Statistical analysis

The normality of each dataset was determined using the Kolmogorov–Smirnov’s, Anderson–Darling’s, D’AgostinoPearson’s, and Shapiro–Wilk’s tests, and the quantile–quantile (q–q) plot. For non-parametric datasets, the Wilcoxon matched-pairs signed rank test was used to analyse paired data sets, Mann–Whitney for unpaired datasets, and Kruskal–Wallis test for multivariable datasets. For parametric datasets, a paired or unpaired *t*-test was used to analyse data and an ordinary one-way ANOVA was used for multivariable datasets. For parametric datasets where variance differed between groups, unpaired *t*-test with Welch’s correction was used. For non-parametric datasets where SD differed between groups, a log transformation to achieve normal distribution and tests for parametric data were used. Violin plots display the median, along with the 25th and 75th percentiles. Bar plots graphs are expressed as the mean ±standard error of the mean. Graphing of data and analysis of statistical significance was performed in Prism (GraphPad v9) and R (version v4.2.2). Results were considered significant at *P < 0.05, **P < 0.01, ***P < 0.001, ****P < 0.0001.

## Results

### Study cohort

Antibody responses to the SARS-CoV-2 vaccine were measured in a cross-sectional cohort of 93 SARS-CoV-2 naive SLE patients. Thirty four patients were recruited pre-pandemic (no vaccine), and 59 patients post vaccination after either 2 or 3^+^ doses of mRNA-based vaccine (Pfizer or Moderna, *n* = 34); non-mRNA vector-based vaccine (Astrazeneca, *n* = 7); or a combination of two vaccine types (*n* = 15). Healthy volunteers (135) were also recruited; 70 pre-pandemic (no vaccine), and 65 after either 2 or 3^+^ doses of mRNA-based vaccine (Pfizer or Moderna, *n* = 57); non-mRNA vector-based vaccine (Astrazeneca, *n* = 3); or a combination of two vaccine types (*n* = 4). A small longitudinal cohort of 18 SLE patients and 22 healthy controls was also included. Further demographic details for the cross-sectional and longitudinal cohorts can be found in Table 1 and Supplementary Table 1, respectively.

**Table 1:** demographics of cross-sectional cohort of SLE patients and healthy controls

	HC (*n* = 65)		SLE (*n* = 59)	
**Dose #**	2 (*n* = 34)	3–4 (*n* = 31)	2 (*n* = 21)	3–4 (*n* = 38)
Age mean	31.9	32.2	42.2	44.9
Age range	20–56	20–58	25–66	28–80
Sex (%F/M)	58.8/41.2	54.8/45.2	90.5/9.5	100/0
**Ethnicity**				
White	47.1	74.2	38.1	55.3
Afro-Caribbean	0.0	0.0	38.1	5.3
South/East Asian	11.8	19.4	23.8	31.6
Other	5.9	6.5	0.0	7.9
No data	35.3	0.0	0.0	0.0
**Treatment**				
HCQ			66.7	71.1
Pred			66.7	63.2
MTX			0.0	2.6
MMF			28.6	23.7
Aza			23.8	28.9
Other			19.0	7.9
No medication			14.3	10.5
**Vaccine type**				
mRNA	91.2	83.9	61.9	55.3
non-mRNA	8.8	0.0	33.3	0.0
Combination	0.0	12.9	4.8	36.8
No data	0.0	3.2	0.0	7.9
**Clinical features**				
GS (range, average)			0–17, 6.2	0–10, 1.7
dsDNA (range, average, not determined)			<0.6–397, 64.4, 0	0.7–326, 41.6, 13.2
C3 (range, average, not determined)			0.6–1.5, 1.0, 0	0.6–1.4, 1.0, 5.3
**BILAG (% A/B/C/D/E)**				
General			0/0/0/81.0/19.0	0/0/0/94.7/5.3
Cutaneous			4.8/9.5/4.8/71.4/9.5	0/0/2.6/89.5/7.9
CNS			4.8/0/0/57.1/38.1	0/2.6/0/55.3/42.1
Musculoskeletal			0/9.5/14.3/66.7/9.5	0/0/13.2/86.8/0
Respiratory			0/0/0/52.4/47.6	0/0/0/63.2/36.8
GI			0/0/0/33.3/66.7	0/0/0/44.7/55.3
Ophthalmological			0/0/0/28.6/71.4	0/0/0/42.1/57.9
Renal			9.5/19.0/4.8/57.1/9.5	0/7.9/18.4/63.2/10.5
Hematological			0/0/57.1/38.1/4.8	0/0/47.4/52.6/0

Aza: azathioprine; BILAG: British Isles Lupus Assessment Group; CNS: central nervous system; dsDNA: double-stranded DNA; GI: gastrointestinal; GS: global score; HCQ: hydroxychloroquine; MMF: mycophenolate mofetil; MTX: methotrexate; Pred: prednisolone.

### SLE patients show reduced serum SARS-CoV-2 IgG antibody response compared to healthy individuals

**Figure 1:**
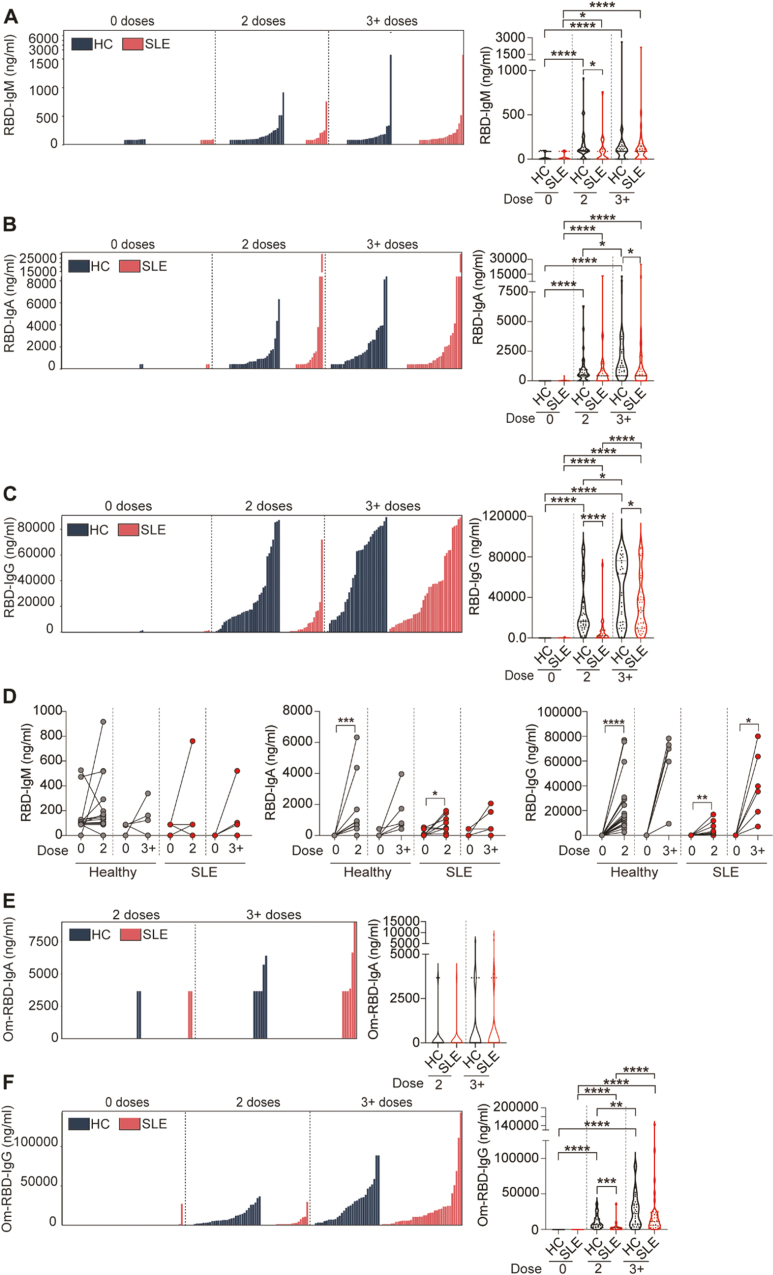
SLE patients have impaired humoral response after SARS-CoV-2 vaccination. (**A-C**) Individual data bar plots and cumulative data violin plots showing the titres of RBD-specific (A) IgM, (B) IgA, and (C) IgG antibodies in a cross-sectional cohort of SLE patients and healthy controls (HC), pre-vaccination and following 2 or 3^+^ doses of vaccine. **(D)** Graphs showing the titres of RBD-specific IgM, IgA, and IgA antibodies in a longitudinal cohort of SLE patients and healthy controls (HC), between pre-vaccination (0 doses) and 2 or 3^+^ doses of vaccine. **(E, F)** Individual data bar plots and cumulative data violin plots showing the titres of Omicron (Om)-specific **(E)** IgA and **(F)** IgG antibodies in a cross-sectional cohort of SLE patients and healthy controls (HC), pre-vaccination and following 2 or 3^+^ doses of vaccine. *P < 0.05, **P < 0.01, ***P < 0.001, ****P < 0.0001, (A–F) by Mann–Whitney of selected group pairs or by Wilcoxon matched-pairs signed rank test. Violin plots display the median, along with the 25th and 75th percentiles

The subunit 1 (S1) of the SARS-CoV-2 spike protein interacts with the ACE receptor on the host cell. This subunit includes the ACE receptor binding domain (RBD). Virus-specific (S1 and RBD) vaccine-derived IgG, IgA, and IgM levels (ng/ml) were assessed using direct ELISA. We report a high degree of variability of vaccine responses for all antibody isotypes in both healthy controls and SLE patients (Fig. 1A–C).

Analysis of specific antibody isotypes showed that IgM responses against both RBD and S1 contributed minimally to the spike-reactivity in healthy and SLE cohorts at any dose tested (Fig. 1A, Supplementary Fig. 1A).

IgA-specific responses to RBD and S1 were impaired in SLE patients after 2 and 3^+^ doses as shown by reduced IgA median titers (IgA titre median after two doses: RBD HC = 527.5 vs SLE = 424.1; S1 HC = 846.5 vs SLE = 0; IgA titre median after 3^+^ doses: RBD HC = 1145 vs SLE = 437.1; S1 HC = 1613 vs SLE = 999.7) (Fig. 1B Supplementary Fig. 1B).

Although the titers of anti-RBD and anti-S1 IgG significantly increased following booster doses (3^+^ doses) in the majority of SLE patients compared to the levels detected after the second dose, median levels remained significantly lower than those in healthy individuals (IgG titre median after 3^+^ doses: RBD HC = 63k vs SLE = 26.7k; S1 HC = 113.7k vs SLE = 53.5k) (Fig. 1C, Supplementary Fig. 1C).

SLE patients are known to present hyper-globulinemia [14] and treatment-derived hypo-globulinemia [15], which might influence vaccine responses. To consider the “weight” that this defect in total antibodies might have on the vaccine-specific Ig response, we calculated the relative levels of antigen-specific IgM/A/G as a percentage of respective total Ig isotype. SLE patients present lower amount of total IgG and IgM and an increase in total IgA levels compared to healthy controls both before and after vaccination (Supplementary Fig. 2A). When calculated as percentage of the total respective Ig isotype, SLE patients continued to show lower RBD-IgG and IgM responses compared to healthy controls after 2 and 3^+^ doses (Supplementary Fig. 2B and D). No significant differences were observed in the percentages of RBD-IgA/total IgA between SLE patients and healthy controls at any of the doses tested (Supplemental Fig. 2C).

Analysis of the longitudinal cohort confirmed that although the levels of RBD-specific IgA and IgG were significantly increased in SLE patients after vaccination, the medians of IgG and IgA titres were higher in healthy compared to SLE patients even after 3^+^dose (titre median after 3^+^ doses: RBD IgG HC = 74.2k vs SLE = 37.5k; IgA HC = 704 vs SLE = 212.1) (Fig. 1D). No changes in IgM levels were recorded after vaccination in these samples (Fig. 1D).

Several studies have indicated that immunosuppressant-drugs and or severity of disease are associated with weaker SARS-CoV-2 vaccination responses [9, 11]. No significant associations with antibody response across different treatments (DMARDs, including hydroxychloroquine (HCQ), or prednisolone) were observed at any of the doses tested (Supplementary Fig. 3A and B). We report that RBD-IgM titres were increased in patients with active disease (BILAG global scoring ≥5) compared to those with inactive disease (BILAG <5) after 3^+^ doses. No associations were found between disease activity and RBD-IgA nor RBD-IgG titres (Supplementary Fig. 3C).

SARS-CoV-2 vaccination targeting the Wuhan-1 (Wu-1) strain has demonstrated limited efficacy against the Omicron variant in healthy individuals [16], however, the ability of SLE patients to respond to new variants remains largely unexplored. Omicron RBD-specific IgA (Om-RBD-IgA) titres were undetectable in the majority of SLE patients and healthy controls for any number of doses (Fig. 1E). We confirm that IgG titres against the Omicron RBD were generally lower compared to the response against the original strain of SARS-CoV-2 after 2 doses in both healthy controls and SLE patients (Fig. 1F). A moderate increase of IgG titres was seen in both groups after 3^+^ doses with healthy controls showing a higher median (Fig. 1F, IgG titre median after 3^+^ doses: Om-RBD HC = 22.2k vs SLE = 12.2k).

### SLE patients exhibit impaired neutralizing capacity against variants of concern.

**Figure 2:**
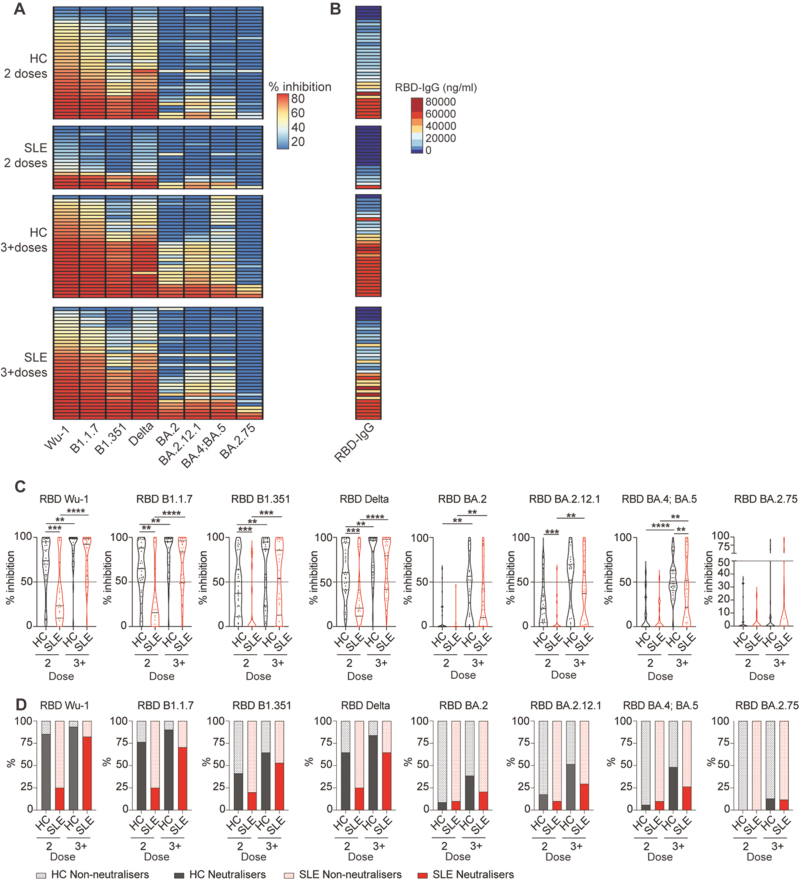
SLE patients show reduced neutralizing capacity against SARS-CoV-2 variants of concern after initial vaccination. **(A)** Heatmap showing percentage inhibition values against the RBD of different SARS-CoV-2 strains (Wu-1, B1.1.7, B1.351, Delta, BA.2, BA.2.12.1, BA.4; BA.5, and BA.2.75) of serum isolated from a cross-sectional cohort of SLE patients and healthy controls (HC), after 2 or 3^+^ doses of vaccine. **(B)** Heatmaps showing the titres of RBD-specific IgG for the corresponding individuals in (A). **(C)** Cumulative data violin plots showing percentage inhibition values against all tested different SARS-CoV-2 strains (Wu-1, B1.1.7, B1.351, Delta, BA.2, BA.2.12.1, BA.4; BA.5, and BA.2.75) of serum isolated from a cross-sectional cohort of SLE patients and healthy controls (HC), after 2 or 3^+^ doses of vaccine. Dotted line on graph indicates the neutralization threshold (50%). **(D)** Stacked bar plots showing the percentage of neutralizers for all tested different SARS-CoV-2 strains (Wu-1, B1.1.7, B1.351, Delta, BA.2, BA.2.12.1, BA.4; BA.5, and BA.2.75) within each group. **P < 0.01, ***P < 0.001, ****P < 0.0001, by (C) by Mann–Whitney of selected group pairs or by Wilcoxon matched-pairs signed rank test. Violin plots display the median, along with the 25th and 75th percentiles

Next, we assessed the functional neutralization capacity of circulating antibodies to block the RBD protein of various SARS-CoV-2 variants from binding to the ACE2 receptor by using an MSD assay in a cross-sectional cohort (SLE *n* = 59, HC *n* = 65) (demographic details in Supplementary Table 2). The analysis included the original strain (Wu-1) and several variants of concern (VOC), comprising Alpha (B.1.1.7), Beta (B.1.351), Delta, and a range of sub-Omicron lineages (BA.2, BA.4, BA.5, BA.2.12.1, and BA.2.75). Reflecting the lower amount of anti-IgG production, viral neutralization, and ratio of neutralizer individuals against all variants were very low in SLE patients after two doses (Fig. 2A–D). Despite lower levels of antibodies detected in SLE patients compared to healthy controls after 3^+^ doses (represented here as alignment between serum IgG levels and respective neutralisation capacity in Fig. 2B), the inhibition capacity for all but one (BA.4;BA.5) strain was not significantly different from healthy controls (Fig. 2C). However, when taking into account the ratio of neutralising individuals, SLE patients showed a lower percentage of neutralisers for all strains after 3^+^ doses (HC = 93.55% vs SLE = 82.35% for Wu-1, HC = 90.32% vs SLE = 70.59% for B.1.1.7, HC = 64.52% vs SLE = 52.94% for B.1.351, HC = 83.87% vs SLE = 64.70% for Delta, HC = 38.71% vs SLE = 20.59% for BA.2, HC = 51.61% vs SLE = 29.41% for BA2.12.1 and HC = 48.39% vs SLE = 26.47% for BA.4/BA.5), but for the strain BA.2.75 (HC = 12.9% vs SLE = 11.76%) (Fig. 2D). Thus, our results reinforce the need for booster doses to increase neutralizing capacity, mainly driven by IgG titres, against VOC in SLE patients to levels comparable to healthy controls.

### SARS-CoV-2-specific B-cell isotype switching is impaired in SLE patients

**Figure 3:**
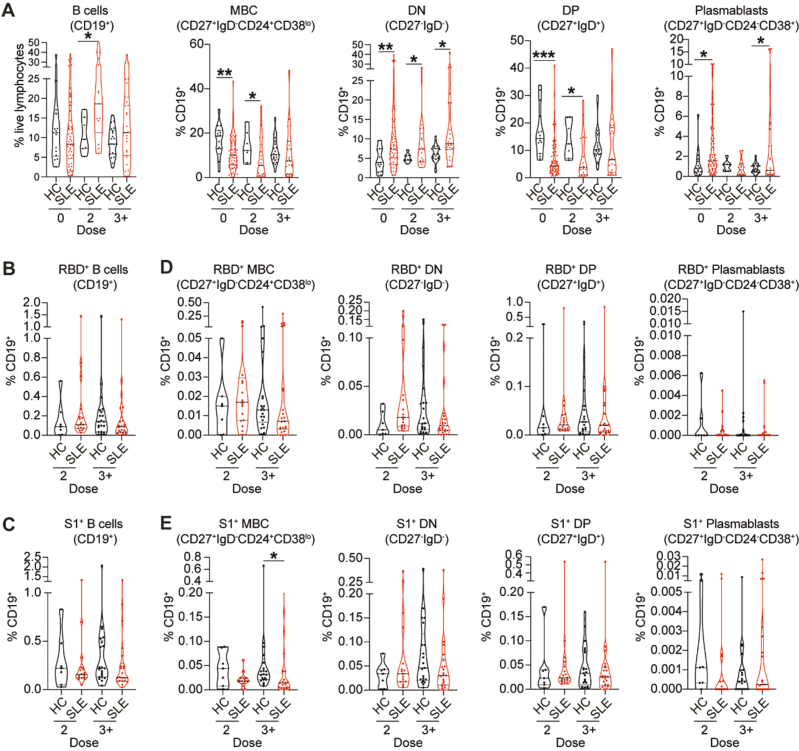
SLE patients show no differences in the frequencies of SARS-CoV-2-specific B cell subsets compared to healthy individuals. **(A)** Cumulative data violin plots showing frequencies of total B cells (CD19^+^), memory B cells (MBC, CD27^+^IgD^−^CD24^+^CD38^lo^), double-negative B cells (DN, CD27^−^IgD^−^), double-positive B cells (DP, CD27^+^IgD^+^) and plasmablasts (CD27^+^IgD^−^CD24^−^CD38^+^) within the total CD19^+^B cell population in *ex vivo* peripheral blood of healthy controls (HC) and SLE patients, pre-vaccination (0 dose) or after 2 or 3^+^ doses of vaccine. **(B,C)** Cumulative data violin plots showing frequencies of total RBD^+^B cells (B) and S1^+^B cells (C) in *ex vivo* peripheral blood of healthy controls (HC) and SLE patients, pre-vaccination (0 dose) or after 2 or 3^+^ doses of vaccine. **(D, E)** Cumulative data violin plots showing frequencies of RBD^+^ (D) and S1^+^ (E) MBC, DN B cells, DP B cells, and plasmablasts, within the total CD19^+^B cell population in *ex vivo* peripheral blood of HC and SLE patients, pre-vaccination (0 dose), or after 2 or 3^+^ doses of vaccine. (*P < 0.05, **P < 0.01 ***P < 0.001, ****P < 0.0001, by unpaired *t*-test, Mann–Whitney test or data transformation according to data distribution and features (see methods). Violin plots display the median, along with the 25th and 75th percentiles

SLE patients present with several inherent B-cell abnormalities including increases in transitional-2, DN B cells, and plasma cells [17–19]. We found increased levels of B cells after 2 and 3^+^ doses in SLE patients compared to healthy individuals (Fig. 3A). We report decreased levels of total memory B cells (MBC; CD27^+^IgD^−^CD24^+^CD38^lo^) and double positive B cells (DP; CD27^+^IgD^+^), mirrored by an increased in double negative B cells (DN; CD27^−^IgD^−^) and plasmablasts (CD27^+^IgD^−^CD24^−^CD38^+^) in SLE patients compared to healthy controls at baseline, differences that were maintained after vaccination (Fig. 3A; gating strategy in Supplementary Fig. 4A and B).

To understand whether changes in B-cell subsets frequency observed in SLE patients, impacted the response to SARS-CoV-2 vaccination, we measured RBD and S1-specific memory B cells, DN, DP, and plasmablasts frequencies in SLE patients and healthy controls (demographics in Supplementary Table 3; gating strategy in Supplementary Fig. 4A and B). There was no difference in the frequencies of total RBD^+^ and S1^+^ B cells between SLE patients and healthy controls (Fig. 3B and C). Analysis of antigen-specific B-cell subsets, amongst total B cells, show that the frequencies of RBD^+^ and S1^+^ MBCs in SLE patients were decreased (a trend for RBD and significant for S1) compared to healthy controls even after 3^+^ doses of vaccine (Fig. 3D and E). We did not observe any differences between healthy controls and SLE patients in the frequencies of RBD^+^ or S1^+^ DN B cells, DP B cells or plasmablasts (Fig. 3D and E).

**Figure 4:**
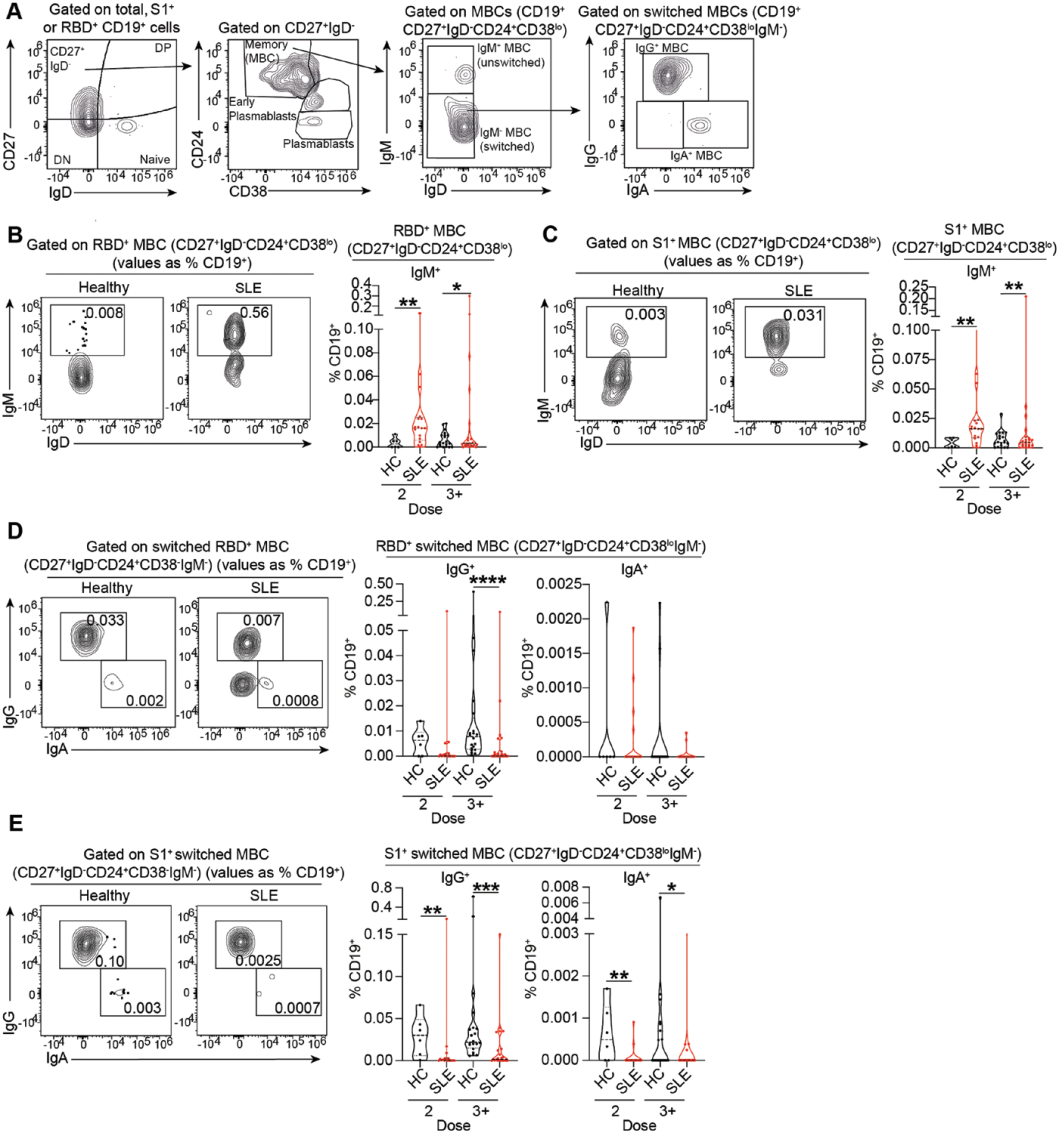
SARS-CoV-2-specific B cells show lower IgG and IgA class-switching in SLE patients. **(A)** Representative contour plots showing the gating strategy for identification of memory B cells (MBCs; CD27^+^IgD^−^CD24^+^CD38^lo^), early plasmablasts (CD27^+^IgD^−^CD24^mid^CD38^+^), and plasmablasts (CD27^+^IgD^−^CD24^−^CD38^hi^), within RBD^+^ and S1^+^ B-cell subsets. IgM^+^ unswitched (CD27^+^IgD^−^CD24^+^CD38^lo^IgM^+^) and IgG^+^ switched (CD27^+^IgD^−^CD24^+^CD38^lo^IgM^−^IgG^+^) or IgA^+^ switched (CD27^+^IgD^−^CD24^+^CD38^lo^IgM^−^IgA^+^) MBCs were identified within RBD^+^ and S1^+^ B-cell subsets. **(B, C)** Representative contour plots and cumulative data violin plots showing the frequencies of (B) RBD-specific and (C) S1-specific IgM^+^ unswitched MBCs (CD27^+^IgD^−^CD24^+^CD38^lo^IgM^+^) in *ex vivo* peripheral blood of healthy controls (HC) and SLE patients, after 2 or 3^+^ doses of vaccine. Values in gates indicate the percentage within CD19^+^ cells. **(D, E)** Representative contour plots and cumulative data violin plots showing the frequencies of (D) RBD-specific and (E) S1-specific IgG^+^ and IgA^+^ switched MBCs (CD27^+^IgD^-^CD24^+^CD38^lo^IgM^−^ IgG^+^/IgA^+^), in *ex vivo* peripheral blood of healthy controls (HC) and SLE patients after 2 or 3^+^ doses of vaccine. Values in gates indicate the percentage within CD19^+^ cells. *P < 0.05, **P < 0.01 ***P < 0.001, ****P < 0.0001, by Mann–Whitney test or data transformation according to data distribution and features (see methods). Violin plots display the median, along with the 25th and 75th percentiles

Next, we assessed how repeated vaccinations affect the frequencies of memory unswitched (IgM^+^) or memory switched (IgG^+^/IgA^+^) RBD^+^ and S1^+^ B cells (Fig. 4A). There was an increased frequency of RBD^+^/S1^+^ IgM^+^ unswitched MBCs following 2 and 3^+^ doses in SLE patients (Fig. 4B and C), and an increase in and RBD^+^/S1^+^ IgM^+^ DN B cells after two doses (Supplementary Fig. 4C and D). This was accompanied by a decrease in RBD^+^/S1^+^ IgG^+^ MBCs (Fig. 4D and E), and RBD^+^/S1^+^ IgG^+^ DN B cells (Supplementary Fig. 4C and D) after 2 or 3^+^ doses in SLE patients compared to healthy controls. RBD^+^ and S1^+^ IgG^+^ memory switched B-cell frequencies significantly correlated with RBD- and S1-IgG titres for HC but failed to correlate for SLE patients (Supplementary Fig. 4G). We also reported increased frequencies of RBD^+^/S1^+^ IgM^+^ early plasmablasts (early-PB) (CD27^+^IgD^-^CD24^mid^CD38^+^) in SLE patients compared to healthy controls (Supplementary Fig. 4E and F), previously shown to arise from extrafollicular responses [17–19]. When stratifying SLE patients into responders and non-responders according to their RBD- and S1-IgG levels, we found increased frequencies of IgM^+^ early-PB in the non-responder group, compared to the responder group (Supplementary Fig. 4H).

The results for RBD^+^IgA^+^ MBCs were difficult to interpret due to very low frequencies of RBD^+^IgA^+^ B cells (Fig. 4D). Nevertheless, S1^+^IgA^+^MBCs were significantly reduced in SLE patients after 2 and 3^+^ doses, compared to healthy controls (Fig. 4E). No differences were observed between SLE patients and healthy controls in the frequencies of IgA^+^DN B cells for both RBD^+^ /S1^+^ and populations (Supplementary Fig. 4C and D).

The accumulation of antigen-specific IgM^+^ early-PB in SLE patients suggests a defect in CSR supported by an extrafollicular differentiation pathway. We calculated the ratio between RBD^+^/S1^+^ switched (IgA or IgG) MBCs and RBD^+^/S1^+^ IgM^+^ early-PB as an index (CSR ratio) of efficient CSR compared to extrafollicular responses. SLE patients showed a significantly lower CSR ratio compared to healthy controls (Supplementary Fig. 5A and B). A similar CSR impairment was also observed in non-antigen-specific (S1^−^) B cells (Supplementary Fig. 5C–F), suggesting that this defect may not be limited to vaccine-derived antigen-specific B cells.

### IFNα impairs class-switch recombination *in vitro* and promotes IgM^+^ early plasmablasts

Our findings and those from others [20] suggest that SLE patients exhibit impaired CSR and increased frequencies of vaccine-specific IgM^+^ MBCs and IgM^+^ early-PBs. Given the pivotal role of IFNα in SLE pathogenesis and in driving plasma cell differentiation [21, 22], we hypothesized that exposure to elevated IFNα levels may contribute to the observed CSR imbalance in SLE patients.

**Figure 5:**
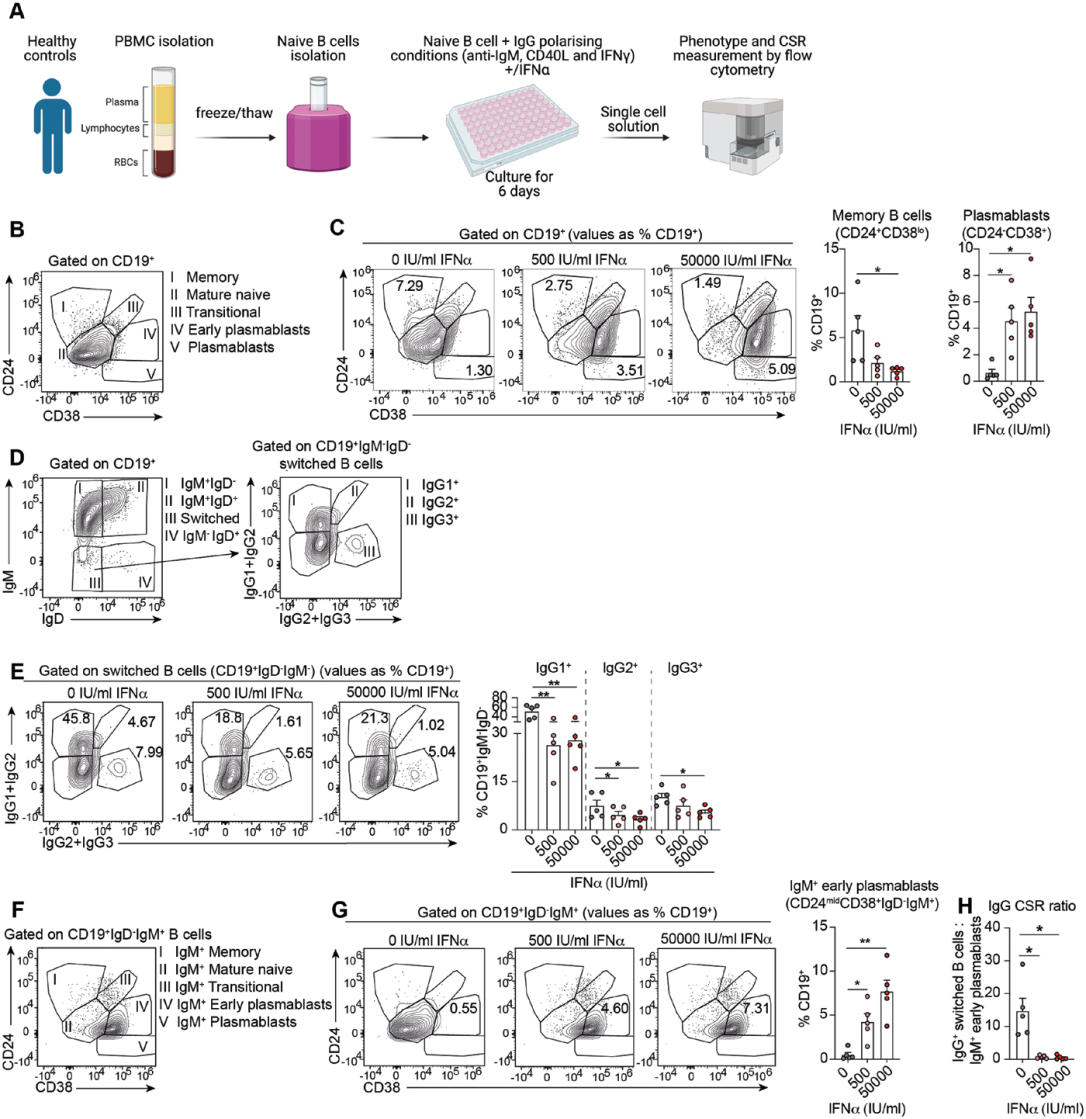
interferon alpha suppresses healthy naive B-cell class switching to IgG. (**A)** Schematic of class-switching *in vitro* experiment. Naive B cells (CD27^−^IgD^+^) were isolated from previously frozen PBMCs of healthy controls. Isolated naive B cells were cultured under IgG class switching polarizing conditions (anti-IgM, CD40L, and IFNγ) together with 0, 500, or 50 000 IU/ml of IFNα for 6 days. After 6 days of culture, cells were harvested and stained for flow cytometry to assess their phenotype and CSR status. *Created in BioRender. Mauri, C. (2025)* *https://BioRender.com/h46s057*. **(B)** Contour plots showing the gating strategy for the identification of memory B cells (CD19^+^CD24^+^CD38^lo^), mature naive B cells (CD19^+^CD24^mid^CD38^mid^), transitional B cells (CD19^+^CD24^+^CD38^+^), early plasmablasts (CD19^+^CD24^mid^CD38^+^), and plasmablasts (CD19^+^CD24^−^CD38^+^) after 6 days in IgG-polarizing class-switch stimulation of isolated healthy naive B cells (CD27^−^IgD^+^). **(C)** Representative contour plots and cumulative data bar graphs showing the frequencies of memory B cells (CD19^+^CD24^+^CD38^lo^) and plasmablasts (CD19^+^CD24^−^CD38^+^) after 6 days stimulation of isolated healthy naive B cells under IgG-polarizing conditions and increasing concentrations of recombinant IFNα. Values in gates indicate the percentage within CD19^+^ cells. **(D)** Contour plots showing the gating strategy for the identification of IgG1^+^, IgG2^+^ and IgG3^+^ switched B cells after 6 days of stimulation of isolated healthy naive B cells under IgG-polarising conditions. **(E)** Representative contour plots and cumulative bar graphs showing the frequencies of IgG1^+^, IgG2^+,^ and IgG3^+^ switched B cells, after 6 days of stimulation of isolated healthy naive B cells under IgG-polarizing conditions and increasing concentrations of recombinant IFNα. Values in gates indicate the percentage within CD19^+^IgD^−^IgM^−^ cells. **(F)** Contour plots showing the gating strategy for the identification of IgM^+^ memory, IgM^+^ mature naive, IgM^+^ transitional B cells, IgM^+^ early plasmablasts, and IgM^+^ plasmablasts after 6 days in IgG-polarizing class-switch stimulation of isolated healthy naive B cells. **(G)** Representative contour plots and cumulative bar graphs showing the frequencies of IgM^+^ early plasmablasts (CD19^+^CD24^mid^CD38^+^ IgD^-^IgM^+^) after 6 days stimulation of isolated healthy naive B cells under IgG-polarizing conditions and increasing concentrations of recombinant IFNα. Values in gates indicate the percentage within CD19^+^ cells. **(H)** Cumulative data bar graph showing the ratio between IgG^+^ switched memory B cells (sum of IgG1^+^, IgG2^+,^ and IgG3^+^ frequencies within CD19^+^IgD^−^IgM^−^ cells) and IgM^+^ early plasmablasts, after 6 days stimulation of isolated healthy naive B cells under IgG-polarizing conditions and increasing concentrations recombinant IFNα. *P < 0.05, **P < 0.01, by Kruskal–Wallis test with Dunn’s multiple comparison. Error bars in bar plots show mean±SEM

We took advantage of a previously established culture system whereby healthy naive B cells were stimulated *in vitro* for 6 days with a combination of anti-IgM, CD40L, and IFNγ to induce class-switching to IgG isotypes [23]. Increasing concentrations of IFNα were added to the culture system to model the potential effect of IFNα on B-cell differentiation and CSR of SLE B cells (Fig. 5A). As expected, IFNα reduced memory B cells while promoting an increase of plasmablasts *in vitro* (Fig. 5B and C). In addition, high IFNα levels reduced switching of healthy naive B cells to IgG1, IgG2, and IgG3, under CSR-IgG-polarising conditions (Fig. 5D and E). Supporting our *ex vivo* findings in SLE patient B cells, we observed a significant increase of IgM^+^ early-PB frequencies and a decreased ratio of IgG^+^ B cells to IgM^+^ early-PBs (CSR ratio) under these polarizing *in vitro* conditions (Fig. 5F–H). Together this *in vitro* modelling of SLE B-cell CSR indicates that high concentrations of circulating IFNα in SLE patients may contribute to the impaired CSR of B cells and subsequently result in weaker antibody responses following SARS-CoV-2 vaccination.

## Discussion

The efficacy of the SARS-CoV-2 vaccination programme has been well-documented in non-immunocompromised cohorts, however, our understanding of how SLE patients respond to new vaccines remains unknown. Here we show that SLE patients failed to generate robust SARS-CoV-2-specific protective antibody responses after two doses, including vaccine-specific IgG and IgA, and neutralizing capacity against the original strain of the virus and VoC. We demonstrate that booster vaccination provides sufficient protection in around three-quarters of SLE patients compared to the levels of protection reached in healthy individuals. Others have reported similar findings, as two-thirds of SLE patients treated with belimumab seroconverted only after booster vaccination [24]. Booster vaccination also improved RBD-IgG responses in an adolescent SLE cohort and enabled seroconversion against Omicron variants [25]. It should be noted that even with booster vaccination many SLE patients did not acquire sufficient humoral protection, particularly against several VoC. These observations highlight the need for tailored vaccination strategies for SLE patients and offer important lessons for future pandemics.

The reduced humoral protection observed in SLE patients after initial SARS-CoV-2 vaccination has been previously linked to immunosuppressant drugs [7, 26]. However, there is no clear consensus on whether specific SLE treatments impair vaccine efficacy, and current research [27], including our study, shows variable findings. The impaired efficacy of SARS-CoV-2 vaccination may also be the consequence of SLE disease activity, as we observed higher RBD-IgM responses in patients with active disease. Nevertheless, the variability observed between patients suggests that multiple factors may contribute to the reduced vaccine responses in SLE patients.

We provide novel insight by reporting that vaccine-specific memory and DN B cells in SLE patients express IgM without class-switching to IgG or IgA. Additionally, SLE patients present with increased frequencies of vaccine-specific IgM^+^ early plasmablasts. It is tempting to speculate that SARS-CoV-2 vaccine responses in SLE patients occur extrafollicularly. Extrafollicular reactions typically have lower CSR levels and rapid IgM^+^ plasmablast generation compared to follicular and GC-dependent B-cell responses [28]. It has been suggested that extrafollicular reactions provide reduced vaccine efficacy. Indeed, expansions of DN B cells have been linked to reduced SARS-CoV-2 neutralizing responses and lower frequencies of RBD-specific B cells [29]. Notably, extrafollicular DN B cells highly express autoimmune regulator (AIRE), and a recent preprint demonstrated that AIRE in GC B cells suppresses AID-mediated B-cell affinity maturation and CSR [30]. Whether AIRE expression in extrafollicular B-cell responses contributes to the impaired CSR observed in SLE patients will be subject of our future investigation.

Finally, we demonstrated that *in vitro* stimulation of healthy B cells with IFNα recapitulated the reduced IgG CSR and expanded IgM^+^ early plasmablast population seen in SLE patients *ex vivo*. IFNα may contribute to reduced SARS-CoV-2 vaccine responses in SLE patients. High levels of serum IFNα and upregulation of interferon-inducible genes are associated with active SLE [21, 22], and we identified that clinically active patients present with increased RBD-IgM responses compared to inactive patients. IFNα signalling is also shown to promote extrafollicular B-cell reactions [28, 31, 32]. The exact impact of IFNα on SARS-CoV-2 vaccine responses in SLE patients remains unclear and warrants further study.

There are limitations to this study. Our cohort of longitudinal SLE patients was small, as the majority of SLE patients were shielding during the initial vaccination programme. We were also only able to analyse a small number of patients treated with DMARDs, and other drugs included in the study, which prevent us from drawing conclusions about the potential effect of individual medications or dosage on vaccine efficacy.

In summary we report that although repeated doses of vaccine improve protective responses in SLE patients, they remain significantly lower than those achieved in healthy individuals. We also report a skewed memory B cell formation towards an accumulation of unswitched IgM^+^ memory B cells. As such, continued booster and optimization of vaccination strategy should be considered for patients with SLE.

## Supplementary data

Supplementary data is available at *Clinical and Experimental Immunology* online.

uxae119_suppl_Supplementary_Tables_S1-S3_Figures_S1-S5

## Data Availability

Data are available upon request.

## Study conception and design

Mauri, Montamat, Bradford.

## Acquisition of data

Montamat, Meehan, Yıldırım, Guimarães, Goldblatt, Johnson, Isenberg.

## Analysis and interpretation of data

Mauri, Montamat, Meehan, Bradford.

## References

[1] Hurtado C, Rojas-Gualdrón DF, Urrego R, Cashman K, Vásquez-Trespalacios EM, Díaz-Coronado JC, et al Altered B cell phenotype and CD27+ memory B cells are associated with clinical features and environmental exposure in Colombian systemic lupus erythematosus patients. Front Med (Lausanne) 2022, 9, 950452. doi: https://doi.org/10.3389/fmed.2022.950452 36148466 PMC9485945

[2] Mauri C, Menon M. Human regulatory B cells in health and disease: therapeutic potential. J Clin Investig 2017, 127, 772–9. doi: https://doi.org/10.1172/JCI85113 28248202 PMC5330739

[3] Jones DD, Wilmore JR, Allman D. Cellular dynamics of memory B cell populations: IgM+ and IgG+ memory B cells persist indefinitely as quiescent cells. J Immunol 2015, 195, 4753–9. doi: https://doi.org/10.4049/jimmunol.1501365 26438523 PMC4637268

[4] Mesin L, Ersching J, Victora GD. Germinal center B cell dynamics. Immunity 2016, 45, 471–82. doi: https://doi.org/10.1016/j.immuni.2016.09.001 27653600 PMC5123673

[5] Roco JA, Mesin L, Binder SC, Nefzger C, Gonzalez-Figueroa P, Canete PF, et al Class-switch recombination occurs infrequently in germinal centers. Immunity 2019, 51, 337–50.e7. doi: https://doi.org/10.1016/j.immuni.2019.07.001 31375460 PMC6914312

[6] Laidlaw BJ, Ellebedy AH. The germinal centre B cell response to SARS-CoV-2. Nat Rev Immunol 2022, 22, 7–18. doi: https://doi.org/10.1038/s41577-021-00657-1 34873279 PMC8647067

[7] Petri M, Joyce D, Haag K, Fava A, Goldman DW, Zhong D, et al Effect of systemic lupus erythematosus and immunosuppressive agents on COVID-19 vaccination antibody response. Arthritis Care Res (Hoboken) 2023, 75, 1878–1885. doi:/10.1002/acr.25094 36714913 PMC10387122

[8] Yuki EFN, Borba EF, Pasoto SG, Seguro LP, Lopes M, Saad CGS, et al Impact of distinct therapies on antibody response to SARS-CoV-2 vaccine in systemic lupus erythematosus. Arthritis Care Res (Hoboken) 2022, 74, 562–71. doi: https://doi.org/10.1002/acr.24824 34806342 PMC9011410

[9] Izmirly PM, Kim MY, Samanovic M, Fernandez-Ruiz R, Ohana S, Deonaraine KK, et al Evaluation of immune response and disease status in systemic lupus erythematosus patients following SARS–CoV-2 vaccination. Arthritis Rheumatol 2022, 74, 284–294. doi:/10.1002/art.41937 34347939 PMC8426963

[10] Sjöwall J, Azharuddin M, Frodlund M, Zhang Y, Sandner L, Dahle C, et al SARS-CoV-2 antibody isotypes in systemic lupus erythematosus patients prior to vaccination: associations with disease activity, antinuclear antibodies, and immunomodulatory drugs during the first year of the pandemic. Front Immunol 2021, 12, 724047. doi: https://doi.org/10.3389/fimmu.2021.724047 34512651 PMC8430325

[11] Moyon Q, Sterlin D, Miyara M, Anna F, Mathian A, Lhote R, et al BNT162b2 vaccine-induced humoral and cellular responses against SARS-CoV-2 variants in systemic lupus erythematosus. Ann Rheum Dis 2022, 81, 575–83. doi: https://doi.org/10.1136/annrheumdis-2021-221097 34607791 PMC8494536

[12] Ammitzbøll C, Bartels LE, Bøgh Andersen J, Risbøl Vils S, Elbæk Mistegård C, Dahl Johannsen A, et al Impaired antibody response to the BNT162b2 messenger RNA coronavirus disease 2019 vaccine in patients with systemic lupus erythematosus and rheumatoid arthritis. ACR Open Rheumatol 2021, 3, 622–8. doi: https://doi.org/10.1002/acr2.11299 34273260 PMC8426741

[13] Amanat F, Stadlbauer D, Strohmeier S, Nguyen THO, Chromikova V, McMahon M, et al A serological assay to detect SARS-CoV-2 seroconversion in humans. Nat Med 2020, 26, 1033–1036. 10.1038/s41591-020-0913-5 PMC8183627 32398876

[14] Cuadrado MJ, Calatayud I, Urquizu-Padilla M, Wijetilleka S, Kiani-Alikhan S, Karim MY. Immunoglobulin abnormalities are frequent in patients with lupus nephritis. BMC Rheumatol 2019, 3, 30. doi: https://doi.org/10.1186/s41927-019-0079-2 31453435 PMC6702722

[15] Lim E, Tao Y, White AJ, French AR, Cooper MA. Hypogammaglobulinemia in pediatric systemic lupus erythematosus. Lupus 2013, 22, 1382–7. doi: https://doi.org/10.1177/0961203313507990 24106215 PMC3840537

[16] Tan CY, Chiew CJ, Pang D, Lee VJ, Ong B, Lye DC, et al Protective immunity of SARS-CoV-2 infection and vaccines against medically attended symptomatic omicron BA.4, BA.5, and XBB reinfections in Singapore: a national cohort study. Lancet Infect Dis 2023, 23, 799–805. doi: https://doi.org/10.1016/s1473-3099(23)00060-9 36924786 PMC10306341

[17] Karrar S, Cunninghame Graham DS. Abnormal B cell development in systemic lupus erythematosus: what the genetics tell us. Arthritis Rheumatol 2018, 70, 496–507. doi: https://doi.org/10.1002/art.40396 29207444 PMC5900717

[18] Iwata S, Tanaka Y. B-cell subsets, signaling and their roles in secretion of autoantibodies. Lupus 2016, 25, 850–6. doi: https://doi.org/10.1177/0961203316643172 27252261

[19] Jenks SA, Wei C, Bugrovsky R, Hill A, Wang X, Rossi FM, et al B cell subset composition segments clinically and serologically distinct groups in chronic cutaneous lupus erythematosus. Ann Rheum Dis 2021, 80, 1190–200. doi: https://doi.org/10.1136/annrheumdis-2021-220349 34083207 PMC8906255

[20] Faliti CE, Anam FA, Cheedarla N, Woodruff MC, Usman SY, Runnstrom MC, et al Poor immunogenicity upon SARS-CoV-2 mRNA vaccinations in autoimmune SLE patients is associated with pronounced EF-mediated responses and anti-BAFF/Belimumab treatment. medRxiv 2023. doi:/10.1038/s41590-024-02010-9

[21] Bradford HF, Haljasmägi L, Menon M, McDonnell TCR, Särekannu K, Vanker M, et al Inactive disease in patients with lupus is linked to autoantibodies to type I interferons that normalize blood IFNα and B cell subsets. Cell Rep Med 2023, 4, 100894. doi: https://doi.org/10.1016/j.xcrm.2022.100894 36652906 PMC9873953

[22] Menon M, Blair PA, Isenberg DA, Mauri C. A regulatory feedback between plasmacytoid dendritic cells and regulatory B cells is aberrant in systemic lupus erythematosus. Immunity 2016, 44, 683–97. doi: https://doi.org/10.1016/j.immuni.2016.02.012 26968426 PMC4803914

[23] Ng JCF, Montamat Garcia G, Stewart AT, Blair P, Mauri C, Dunn-Walters DK, et al sciCSR infers B cell state transition and predicts class-switch recombination dynamics using single-cell transcriptomic data. Nat Methods 2024, 21, 823–34. doi: https://doi.org/10.1038/s41592-023-02060-1 37932398 PMC11093741

[24] Tunitsky-Lifshitz Y, Maoz-Segal R, Niznik S, Shavit R, Haj Yahia S, Langevitz P, et al The third dose of BNT162b2 COVID-19 vaccine is efficacious and safe for systemic lupus erythematosus patients receiving belimumab. Lupus 2023, 32, 675–9. doi: https://doi.org/10.1177/09612033231164262 36952594 PMC10037128

[25] Piyaphanee N, Charuvanij S, Thepveera S, Toh ZQ, Licciardi PV, Pattaragarn A, et al Immunogenicity and safety of BNT162b2 vaccination in adolescents with systemic lupus erythematosus. Lupus 2024, 33, 450–61. doi: https://doi.org/10.1177/09612033241232576 38335115

[26] Garcia-Cirera S, Calvet J, Berenguer-Llergo A, Pradenas E, Marfil S, Massanella M, et al Glucocorticoids’ treatment impairs the medium-term immunogenic response to SARS-CoV-2 mRNA vaccines in systemic lupus erythematosus patients. Sci Rep 2022, 12, 14772. doi: https://doi.org/10.1038/s41598-022-18996-x 36042275 PMC9427088

[27] Saxena A, Guttmann A, Masson M, Kim MY, Haberman RH, Castillo R, et al Evaluation of SARS-CoV-2 IgG antibody reactivity in patients with systemic lupus erythematosus: analysis of a multi-racial and multi-ethnic cohort. Lancet Rheumatol 2021, 3, e585–94. doi: https://doi.org/10.1016/s2665-9913(21)00114-4 34075358 PMC8159192

[28] Al-Aubodah TA, Aoudjit L, Pascale G, Perinpanayagam MA, Langlais D, Bitzan M, et al The extrafollicular B cell response is a hallmark of childhood idiopathic nephrotic syndrome. Nat Commun 2023, 14, 7682. doi: https://doi.org/10.1038/s41467-023-43504-8 37996443 PMC10667257

[29] Yam-Puc JC, Hosseini Z, Horner EC, Gerber PP, Beristain-Covarrubias N, Hughes R, et al; CITIID-NIHR COVID−19 BioResource Collaboration. Age-associated B cells predict impaired humoral immunity after COVID-19 vaccination in patients receiving immune checkpoint blockade. Nat Commun 2023, 14, 3292. doi: https://doi.org/10.1038/s41467-023-38810-0 37369658 PMC10299999

[30] Zhou JZ, Huang B, Pei B, Sun GW, Pawlitz MD, Zhang W, et al A germinal center checkpoint of AIRE in B cells limits antibody diversification. bioRxiv 2024. doi:/10.1101/2024.01.10.574926

[31] Peng SL, Szabo SJ, Glimcher LH. T-bet regulates IgG class switching and pathogenic autoantibody production. Proc Natl Acad Sci U S A 2002, 99, 5545–50. doi: https://doi.org/10.1073/pnas.082114899 11960012 PMC122806

[32] Swanson CL, Wilson TJ, Strauch P, Colonna M, Pelanda R, Torres RM. Type I IFN enhances follicular B cell contribution to the T cell-independent antibody response. J Exp Med 2010, 207, 1485–500. doi: https://doi.org/10.1084/jem.20092695 20566717 PMC2901065

